# The causal relationship between antibody-mediated immune responses to infections and Graves’ disease: a bi-directional Mendelian randomization analysis

**DOI:** 10.3389/ebm.2026.11238

**Published:** 2026-09-16

**Authors:** Xinwei Zhang, Wenyu Xu, Zhou Liu, Jinan Zhang, Xiaorong Yang

**Affiliations:** 1 Graduate School, Shanghai University of Traditional Chinese Medicine, Shanghai, China; 2 Department of Endocrinology and Rheumatology, Shanghai University of Medicine and Health Sciences Affiliated Zhoupu Hospital, Shanghai, China; 3 Department of Obstetrics and Gynecology, Shanghai University of Medicine and Health Sciences Affiliated Zhoupu Hospital, Shanghai, China; 4 Shanghai University of Traditional Chinese Medicine, Shanghai, China

**Keywords:** genome-wide association study, Graves’ disease, immune response, infections, Mendelian randomization

## Abstract

The pathogenesis of Graves’ disease (GD) has not been fully elucidated, and antibody-mediated immune responses following microbial infection may be involved in its development. We conducted a bidirectional two-sample Mendelian randomization analysis using genetic variants associated with GD and 46 antibody-mediated immune-response phenotypes obtained from large-scale genome-wide association studies. In the forward analysis, none of the antibody-mediated immune-response phenotypes was significantly associated with GD risk after Bonferroni or false discovery rate (FDR) correction. Three Epstein–Barr virus antibody phenotypes showed nominal inverse associations with GD under the inverse-variance weighted method, but these findings did not survive multiple-testing correction and showed evidence of heterogeneity, with additional evidence of horizontal pleiotropy for the ZEBRA antibody phenotype. In the reverse analysis, several associations survived multiple-testing correction, although sensitivity analyses indicated heterogeneity or horizontal pleiotropy for the corrected associations except anti-polyomavirus 2 IgG seropositivity. After jointly considering multiple-testing correction and sensitivity analyses, genetic liability to GD was associated with higher anti-polyomavirus 2 IgG seropositivity (OR = 1.156, 95% CI: 1.050–1.273; P = 0.003), which remained significant after FDR correction but did not reach the Bonferroni-corrected threshold. Four additional associations—lower cytomegalovirus pp52 and pp150 antibody levels and higher *Helicobacter pylori* UreA antibody levels and anti-Merkel cell polyomavirus IgG seropositivity—were nominally significant and should be considered hypothesis-generating. Overall, this bidirectional MR study suggests that genetic liability to GD may influence selected humoral immune-response phenotypes. Notably, the association with anti-polyomavirus 2 IgG seropositivity remained significant after FDR correction, while additional suggestive associations were observed for antibody responses related to CMV, *Helicobacter pylori*, and Merkel cell polyomavirus. These findings provide new genetic insights into the potential relationship between GD and infection-related humoral immunity, while the potential influence of antibody-mediated immune responses on GD risk warrants further investigation.

## Impact statement

Graves’ disease is a common autoimmune thyroid disorder, but the role of infection-related immune responses in its development remains unclear. This study is important because it examines whether antibody responses to common infectious agents are likely to contribute to Graves' disease, or whether Graves’ disease itself may influence these immune responses. By analyzing large genetic datasets in both directions, the study provides evidence that antibody responses to infections may not directly cause Graves’ disease. Instead, genetic susceptibility to Graves’ disease appears to be associated with altered antibody responses to cytomegalovirus, Helicobacter pylori, and polyomaviruses. These findings add new evidence to the literature by shifting attention from infection as a simple trigger toward Graves' disease as a condition that may reshape immune responses to infection. This may help guide future studies on immune monitoring and infection-related care in Graves’ disease.

## Introduction

Graves’ disease (GD) is a common organ-specific autoimmune disorder, a subtype of autoimmune thyroid disease (AITDs), characterized by hyperthyroidism and most commonly seen in women aged 30–60 years [1]. The pathophysiology involves multiple immunoregulatory factors, including immune responses mediated by thyroid autoantibodies. Patients with GD have abnormal stimulatory autoantibody against the thyroid-stimulating hormone receptor (TSHR). These autoantibodies bind to TSHR to activate the adenylate cyclase signaling system, leading to diffuse goiter, hyperthyroidism, and infiltrative ophthalmopathy. The disease reflects a complex interaction between genetic susceptibility, environmental factors, and immune responses, particularly TRAb [2–4]. In recent years, the incidence of this disease has been rising, and the average age of patients is decreasing [5]. The primary treatment modalities include anti-thyroid drugs (ATD), radioactive iodine (RAI), and thyroidectomy. However, these approaches tend to have low remission rates, high recurrence rates, and numerous complications [6, 7].

Antibody-mediated immune response is a key mechanism to defend against infection by pathogenic microorganisms. Many infectious agents may play a role in the onset and progression of noncommunicable diseases (NCDs), especially various autoimmune disorders [8–10]. The onset of AITD may be triggered by infectious agents [11]. Patients with GD might exhibit different response patterns to certain infections due to the persistent high activity of their immune systems. For instance, studies have shown that some patients with GD have positive serum HCV tests [12]. Previous observational and mechanistic studies have suggested that infectious agents may contribute to the development of AITD, although the causal direction and the underlying immune pathways remain uncertain [13].

Mendelian randomization (MR) is a statistical method that uses genetic variants as a tool to assess the causal relationship between risk factors and disease [14]. To uncover the potential causal relationship between the immune response mediated by anti-infective agent antibodies and GD, we conducted a study using two-way Mendelian randomization. Using gene variants as instrumental variable, this method can effectively avoid the confounding factors and reverse causation problems that exist in the traditional observational studies and, thus providing more reliable causal inference. MR avoids the limitations of randomized controlled trials such as ethical limitations, high cost and long duration, and gradually becomes a popular analysis method.

Through this study, we aim to enhance our understanding of the role of antibody-mediated anti-infective immune response in the pathogenesis of GD and explore their potential applications in clinical interventions and treatments for GD.

## Materials and methods

### Study design

This study primarily explores the bidirectional causal relationship between Antibody-Mediated Immune Responses to Infections and Graves’ disease. The research process is illustrated in Figure 1. The MR design relies on three key assumptions: (1) genetic variants are strongly associated with antibody-mediated immune responses to infections and/or GD; (2) these genetic variants are independent of any confounding factors that could affect the outcomes related to antibody-mediated immune responses or GD; and (3) the genetic variants influence GD exclusively through their impact on antibody-mediated immune responses to infections.

**FIGURE 1:**
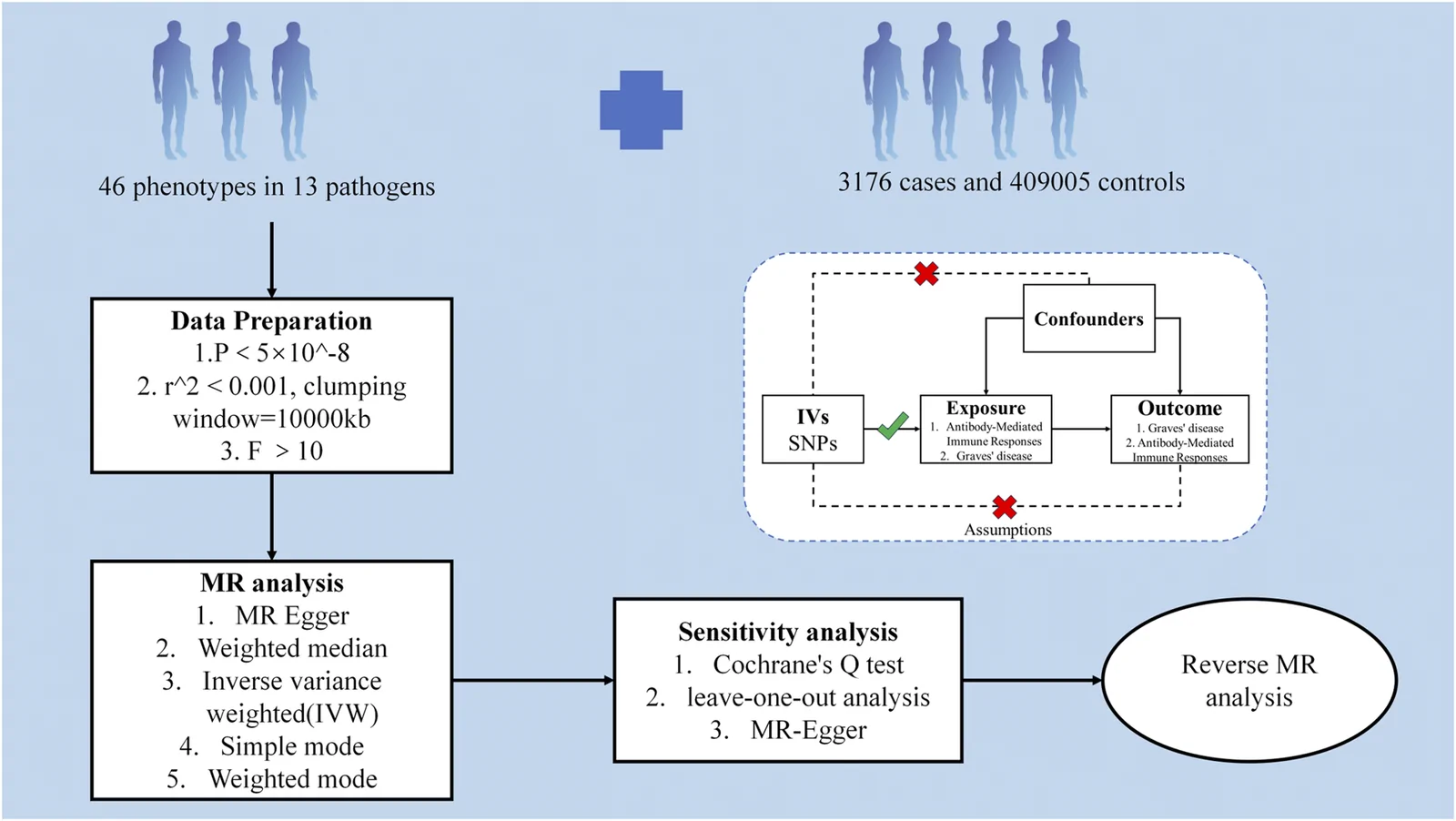
The bidirectional MR design process.

### GWAS summary statistics for antibody-mediated immune responses to infections and Graves’ disease

The data on antibody-mediated immune responses to infections were sourced from a large-scale GWAS study within the UK Biobank. We utilized data concerning 13 pathogens to establish 46 unique phenotypes (GWAS IDs:GCST90006884-GCST90006929). This set comprised 15 case-control phenotypes for seropositivity and 31 phenotypes for quantitative assessments of antibody levels [15]. Detailed information on the GWAS datasets is provided in Supplementary Table S1. The data for GD were derived from the Finnish database^1^ specifically from the finngen_R10_E4_GRAVES_STRICT version, which includes 3,176 cases and 409,005 controls. All data were selected from individuals of European descent.

### Selection of instrumental variables (IVs)

This study uses Graves’ disease (GD) as the outcome variable and employs 46 antibody-mediated immune response phenotypes related to infections as the exposure factors, with corresponding single nucleotide polymorphisms (SNPs) serving as instrumental variables. In the reverse Mendelian Randomization (MR) analysis, the roles of the exposure factors and the outcome variable are inversed, and GD is now used as the exposure factor to investigate its impact on the infection response phenotypes. We use the CLUMP program in PLINK software to set strict filtering criteria. The genome-wide significance threshold was set at 5 × 10^−8^, with an r^2^ threshold of 0.001 and a distance threshold of 10,000 kb [16]. To avoid biases associated with weak instrumental variables, the F-statistic was calculated, setting a cutoff of 10 for the F-value. SNPs with an F-value below this threshold were excluded [17]. The genetic variants ultimately included as instrumental variables in the forward and reverse MR analyses are presented in Supplementary Table S2.

### Statistical analysis

In this study, R4.3.0 was used for statistical analysis, the R software package “TwoSampleMR” was used for basic MR analysis, and the software packages “dplyr”, “circlize” and “ComplexHeatmap” were used for data cleaning and computer-aided mapping. The results of the MR analysis were presented in terms of P-value, odds ratio (OR), and 95% confidence interval (CI). An OR value greater than 1 indicated a positive association between the exposure factor and the outcome, whereas an OR value less than 1 indicated a negative association. When the P value of the MR-egger intercept is greater than 0.05, the inverse variance weighted (IVW) method is more accurate and is the main method of MR analysis [18]. Additionally, to enhance the robustness of the results, we employed methods such as MR-Egger regression and Mendelian Randomization Pleiotropy RESidual Sum and Outlier (MR-PRESSO) [19]. We verified the reliability of our findings through a series of sensitivity analyses, including the exclusion of SNPs with palindromic structures. We also applied the leave-one-out method to sequentially exclude each SNP in the IVW analysis to assess whether the MR results of the remaining SNPs were consistent with the overall findings. The study utilized the Cochran Q test to assess heterogeneity among the SNPs, P-value <0.05 indicated significant heterogeneity. Additionally, the MR-egger intercept was used to detect horizontal pleiotropy, P-value <0.05 indicated the presence of pleiotropy. MR Steiger directionality testing was performed for reverse MR analyses where sufficient information was available to estimate the variance explained in both GD and the antibody-response phenotype. The test was applicable to 31 continuous antibody-level phenotypes but was not performed for 15 binary seropositivity phenotypes because phenotype-specific numbers of seropositive and seronegative participants were unavailable.

## Results

### Exploring the influence of antibody-mediated immune responses on GD

To explore the causal relationship between antibody-mediated immune responses and GD, we used circular heatmaps to illustrate the results of the MR analyses (Figure 2), highlighting the results of five methods: MR Egger, Weighted Median, IVW, Simple Mode, and Weighted Mode.

**FIGURE 2:**
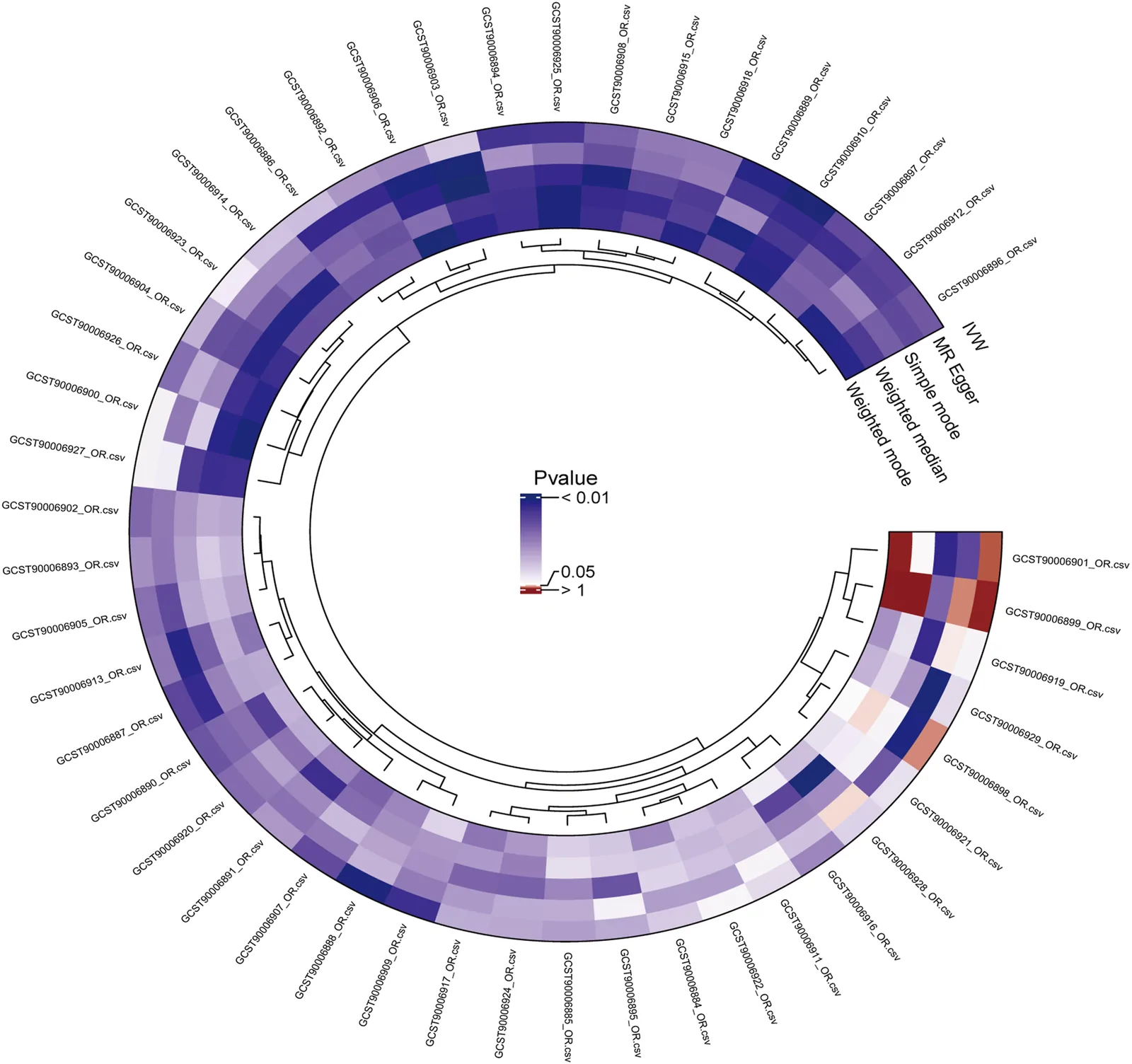
Circular heatmap of forward MR results for 46 antibody-response phenotypes and GD. Each sector represents one phenotype. The five labeled concentric tracks display MR-Egger, weighted median, IVW, simple-mode, and weighted-mode results; color intensity represents the nominal P value according to the displayed scale. The inner dendrogram groups phenotypes by similarity across the five methods. Markers identify IVW associations with raw P < 0.05.

None of the 46 antibody-mediated immune-response phenotypes was significantly associated with GD risk after Bonferroni or FDR correction (Supplementary Table S3). Before multiple-testing correction, the IVW analysis identified nominal inverse associations for three phenotypes: Epstein–Barr virus EA-D antibody levels (OR = 0.552, 95% CI: 0.328–0.928; P = 0.025), Epstein–Barr virus EBNA-1 antibody levels (OR = 0.623, 95% CI: 0.457–0.848; P = 0.003), and Epstein–Barr virus ZEBRA antibody levels (OR = 0.673, 95% CI: 0.491–0.924; P = 0.014) (Figure 3). However, none of these associations survived multiple-testing correction and they should therefore be interpreted as nominal, hypothesis-generating findings.

**FIGURE 3:**
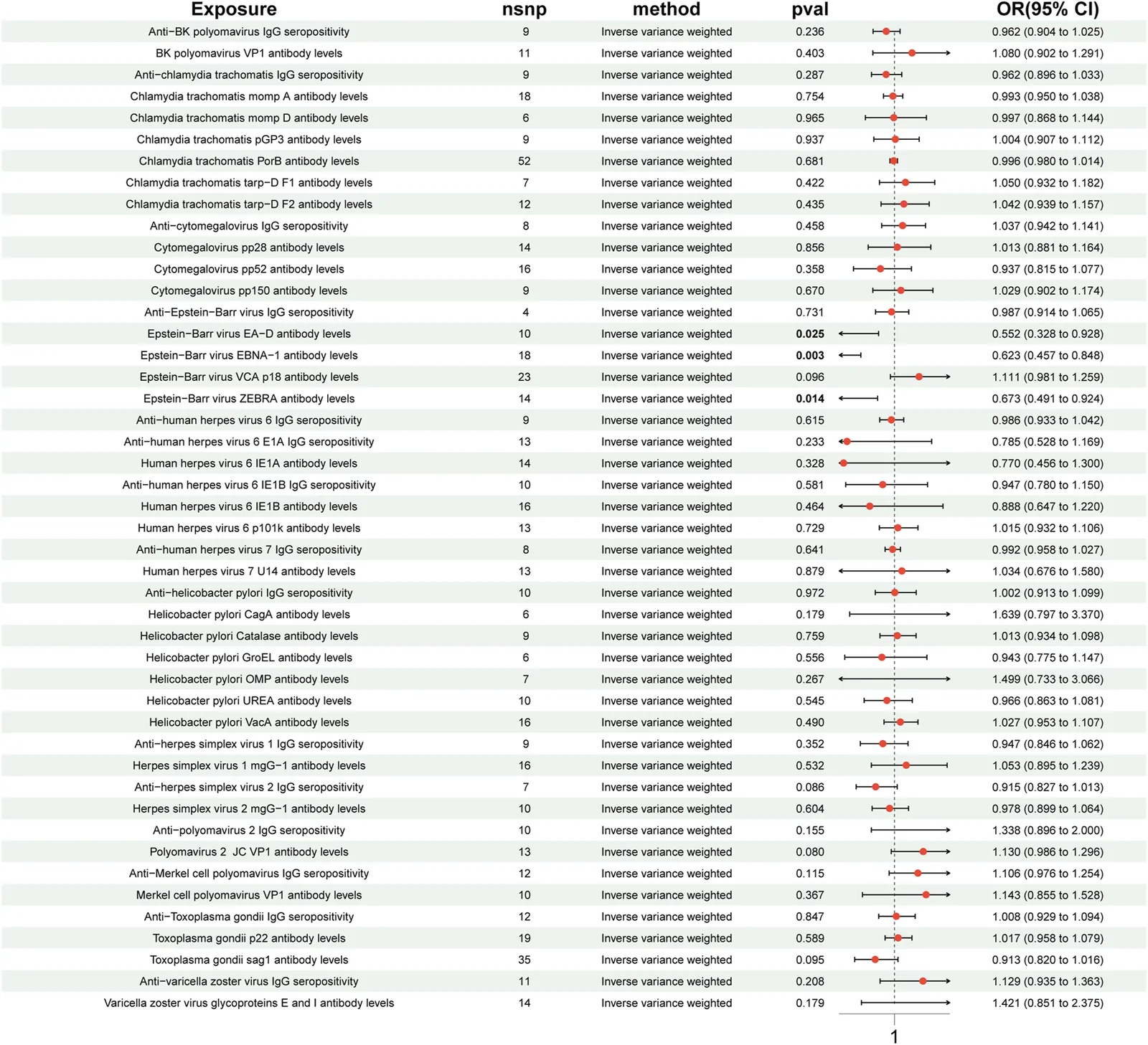
The forest plot of forward MR. Points represent ORs, horizontal lines represent 95% CIs, and the vertical line at OR = 1 represents the null. nsnp is the number of SNP instruments retained after harmonization.

Sensitivity analyses showed evidence of heterogeneity for all three nominal associations, and the corresponding MR-PRESSO global tests were significant. In addition, the MR-Egger intercept indicated evidence of directional horizontal pleiotropy for Epstein–Barr virus ZEBRA antibody levels. These sensitivity-analysis results further suggest that the three nominal forward MR associations should be interpreted cautiously.

### Exploring the influence of GD on antibody-mediated immune responses

In the reverse MR analysis, genetic liability to GD was nominally associated with ten antibody-mediated immune-response phenotypes (Figure 4). After multiple-testing correction across the 46 phenotypes, the association with *Helicobacter pylori* OMP antibody levels survived Bonferroni correction, while associations with Epstein–Barr virus EBNA-1 antibody levels, anti-polyomavirus 2 IgG seropositivity, and anti-varicella zoster virus IgG seropositivity survived FDR correction (Supplementary Table S3). However, sensitivity analyses indicated evidence of heterogeneity or horizontal pleiotropy for some of these associations, including Epstein–Barr virus EA-D, EBNA-1, and ZEBRA antibody levels, *H. pylori* OMP antibody levels, and varicella-zoster virus IgG seropositivity.

**FIGURE 4:**
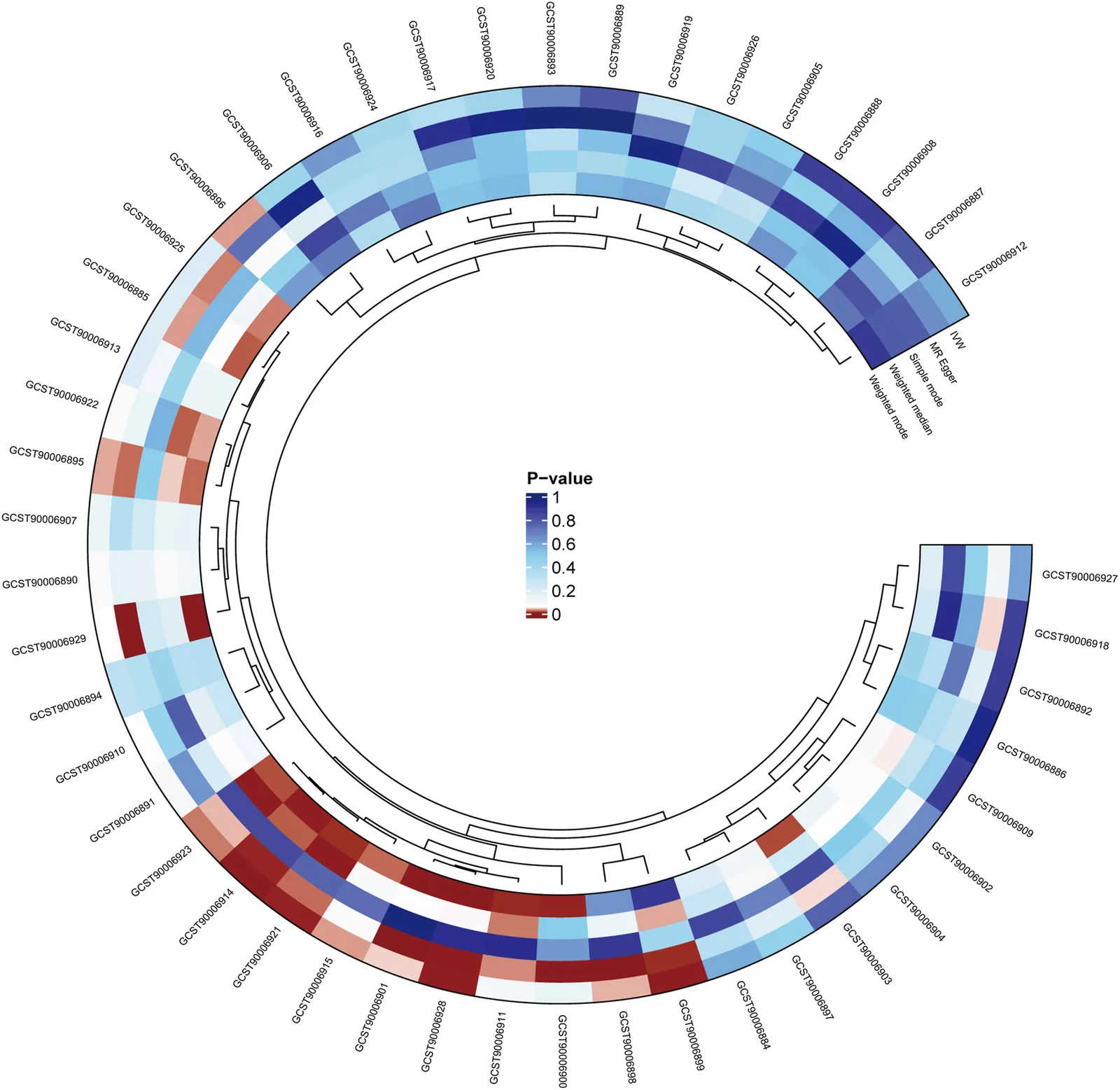
Five methods of reverse MR clustering circos figure. The magnitude of the P-value is indicated by the shade of the color. The five rings show the P values obtained using the IVW, MR-Egger, simple mode, weighted median, and weighted mode methods. Red indicates lower P values, whereas blue indicates higher P values. The inner dendrogram represents hierarchical clustering based on the results of the five MR methods.

After jointly considering multiple-testing correction, heterogeneity, and horizontal pleiotropy, five associations without evidence of substantial violations of the instrumental-variable assumptions were prioritized for further interpretation (Supplementary Tables S4, S5). Genetic liability to GD was associated with lower CMV pp52 antibody levels (OR = 0.951, 95% CI: 0.907–0.996; P = 0.032) and CMV pp150 antibody levels (OR = 0.947, 95% CI: 0.902–0.995; P = 0.030), as well as higher *H. pylori* UreA antibody levels (OR = 1.086, 95% CI: 1.009–1.168; P = 0.029), anti-polyomavirus 2 IgG seropositivity (OR = 1.156, 95% CI: 1.050–1.273; P = 0.003), and anti-Merkel cell polyomavirus IgG seropositivity (OR = 1.107, 95% CI: 1.015–1.208; P = 0.022) (Figure 5). Of these five associations, only that with anti-polyomavirus 2 IgG seropositivity remained significant after FDR correction, although it did not reach the Bonferroni-corrected threshold. The remaining four associations were nominally significant and were therefore considered hypothesis-generating.

**FIGURE 5:**
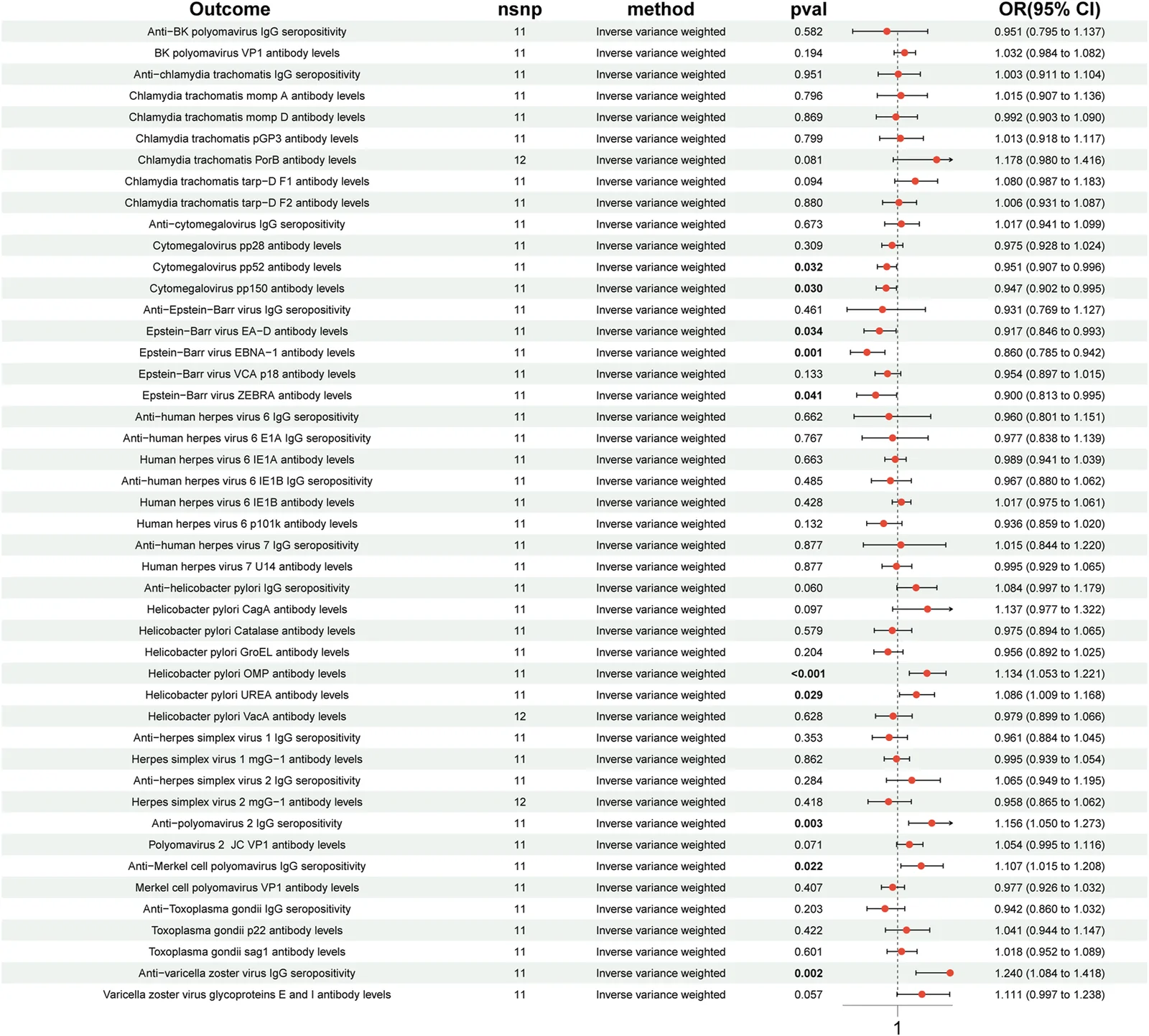
The forest plot of reverse MR. Points represent effect estimates, horizontal lines represent 95% CIs, and nsnp is the number of harmonized SNP instruments.

Steiger directionality testing was applicable to 31 continuous antibody-level phenotypes. For all 31 phenotypes, the variance explained in GD was greater than that explained in the corresponding antibody phenotype, supporting a variance-based direction consistent with GD liability influencing antibody levels. This direction was statistically supported for 29 phenotypes (Steiger P < 0.05). Among the five prioritized associations, Steiger testing supported the proposed direction for the three continuous antibody-level phenotypes. Directionality could not be formally assessed for the two binary seropositivity phenotypes because phenotype-specific numbers of seropositive and seronegative participants were unavailable (Supplementary Table S6).

## Discussion

In the forward MR analysis, none of the infection-related antibody-response phenotypes was associated with GD risk after Bonferroni or FDR correction, although three Epstein-Barr virus antibody phenotypes showed nominal inverse associations. In the reverse MR analysis, genetic liability to GD was associated with selected antibody-response phenotypes. Among the five prioritized reverse associations, the association with anti-polyomavirus 2 IgG seropositivity remained significant after FDR correction, whereas the other four associations were suggestive. These findings should therefore be interpreted at the level of genetically predicted antibody-response traits rather than as evidence of an established immunological mechanism.

Current studies have shown that the development of autoimmune diseases may be related to infection [20–22]. Infection with specific pathogens can trigger or worsen autoimmune responses, especially when combined with genetic predisposition and environmental factors. In some prospective cohort studies and observational studies, immune response to Epstein-Barr virus (EBV) is closely associated with susceptibility to SLE [23], as well as with the pathogenesis and poor control of rheumatoid arthritis (RA) [24]. Elevated levels of EBV EBNA-1 antibodies were also observed in the sera of patients with polymyositis (PM), multiple sclerosis (MS) [25], and Hashimoto’s thyroiditis [26]. HLA class I and immune signaling proteins, such as STAT1 and PKR(which are involved in antiviral responses), have been found to be upregulated in thyroid tissues of GD patients, suggesting that viral infection may initiate or exacerbate the autoimmune process [27]. These studies provide biological context for the relationship between infection and autoimmunity. However, they address a different question from the present MR analysis, which evaluates whether genetically predicted lifelong differences in antibody-response phenotypes are associated with GD risk and whether genetic liability to GD is associated with these phenotypes.

Cytomegalovirus (CMV) is a common DNA virus that lurks in human tissue after infection and can reactivate in cases of immune deficiency, causing herpes [28]. CMV pp52 antibody levels are often used to assess the presence of active CMV infection in the body. Studies have found higher levels of pp52 protein IgG and IgA antibodies against CMV in patients with systemic lupus Erythematosus (SLE) [29]. However, CMV pp52 IgG levels are significantly reduced in patients with multiple sclerosis [30]. Consistent with this observation, our reverse MR analysis showed that genetic liability to GD was nominally associated with lower CMV pp52 antibody levels. These findings suggest that autoimmune disease susceptibility may be associated with alterations in CMV-related humoral immune responses and provide a basis for further investigation. Protein pp150 is related to the maturation and release of CMV and has strong immunogenicity [31]. A study on human cytomegalovirus (hCMV) has shown that anti-PP150 antibodies associated with multiple autoimmune diseases can recognize the viral pp150 protein and the human CIP2A protein, resulting in CD56 (bright) NK cell death [32]. The decrease in the number and function of NK cells is a common pathogenesis of many autoimmune diseases. Some studies have shown that active CMV infection in the synovial fluid of RA patients may exacerbate the inflammatory process. The presence of pp150 also suggests a potential reactivation of CMV in these patients, which could be linked to the pathological processes of rheumatoid arthritis (RA) [31].

*Helicobacter pylori* (*H. pylori*) is a bacterium associated with gastritis, stomach ulcers, and certain types of gastric cancer [33]. In addition, its infection has been linked to various extra-gastric diseases [34], especially the development of AITD, including Hashimoto’s thyroiditis (HT) and GD [21, 35–37]. Studies have shown that antibodies against *H. pylori* can cross-react with thyroid tissue [38]. In one study, researchers used a stool antigen test to analyze *Helicobacter pylori* infection, confirming a significant correlation between this bacterium and patients with GD. In GD patients, the detection rate of Cag-A positive *H. pylori* strain was significantly higher [37]. This may be related to antibodies produced during *Helicobacter pylori* infection, and antibodies against the Cag-A protein can cross-react with thyroid antigen, potentially triggering the development of GD [21]. Urease is a specific enzyme of this bacterium that helps neutralize stomach acid and is closely related to the bacterium’s stress response [39]. There is evidence of common epitopes between thyroid follicular epithelial and gastric parietal cells [40]. Some studies have suggested that proteins from *H. pylori*, including urease, may share epitopes with thyroid follicular cells, potentially leading to molecular mimicry and triggering an autoimmune response [41]. In the present reverse MR analysis, genetic liability to GD was nominally associated with higher *H. pylori* UreA antibody levels. This finding suggests a potential association between GD susceptibility and the H. pylori-related humoral immune response. However, molecular mimicry and antibody cross-reactivity remain possible explanations derived from previous studies and require functional validation in the context of GD.

Polyomavirus (PyVs) is a small DNA virus that causes tumor diseases in humans and other animals [42]. Merkel-cell polyomavirus is known to cause invasive skin cancer of neuroendocrine origin, with a higher risk in immunosuppressed individuals [43]. SV40(Simian virus 40), belonging to the polyomavirus 50 genus, was found in thyroid tissue samples from 20% of GD patients. Although this ratio is low, it suggests that SV40 may be associated with the development of autoimmune thyroid diseases [44]. In our reverse MR analysis, the association between genetic liability to GD and anti-polyomavirus 2 IgG seropositivity remained significant after FDR correction, although it did not reach the Bonferroni-corrected threshold. A nominal association was also observed for anti-Merkel cell polyomavirus IgG seropositivity. These findings support further investigation of the relationship between GD susceptibility and polyomavirus-related antibody responses, while the involvement of viral persistence, reactivation, or thyroid-local infection remains to be established.

Of course, this study has some limitations, First, although the pooled GWAS data of European ancestry were used to reduce ethnic bias, the generalizability of the results across different populations remains uncertain. Further GWAS studies with larger samples are needed to update the results. Second, Only studies of the risk between GD data and antibody-mediated immune responses in the Finnish database have been conducted, and the risk between other GD data and antibody-mediated immune responses in the database has not been explored. Finally, the antibody phenotypes analyzed in this study are proxies for humoral immune responses and may not fully reflect acute pathogen exposure, the timing or reactivation of infection, cellular immunity, or local immune responses within thyroid tissue. Because MR estimates the effects of genetically predicted lifelong differences in these traits, complementary longitudinal and mechanistic studies are needed to further clarify the relationship between infection and GD.

## Conclusion

In summary, our bidirectional MR analysis suggest that antibody-mediated immune responses to infections may not directly cause GD. Instead, the development of GD pathology may be an indirect consequence of these immune responses, or possibly affected by other unknown factors. A deeper understanding of their complex interactions lays the groundwork for further research and potential therapeutic strategies.

## Data Availability

The antibody-response GWAS summary statistics were obtained from the resource reported by Butler-Laporte et al. (2020; DOI: 10.1093/ofid/ofaa450). Summary statistics for GD were obtained from FinnGen R10 (E4_GRAVES_STRICT). All analyses were based on de-identified summary-level data and were conducted in accordance with the data-use conditions of the original repositories.

## Footnotes

1
https://www.finngen.fi/en
